# Effect of Linker Substituent Nature on Performance of Active Sites in UiO-66: Combined FT-IR and DFT Study

**DOI:** 10.3390/ijms241914893

**Published:** 2023-10-04

**Authors:** Viktoriia V. Torbina, Mikhail A. Salaev, Evgeniy A. Paukshtis, Leonarda F. Liotta, Olga V. Vodyankina

**Affiliations:** 1 Laboratory of Catalytic Research, Tomsk State University, 36, Lenin Ave., 634050 Tomsk, Russia; ms.itory@mail.ru (V.V.T.); mihan555@yandex.ru (M.A.S.); 2 Boreskov Institute of Catalysis, SB RAS, 5, Ak. Lavrentieva Ave., 630090 Novosibirsk, Russia; pau@catalysis.ru; 3 Institute for the Study of Nanostructured Materials (ISMN), National Research Council (CNR), 90146 Palermo, Italy

**Keywords:** metal–organic frameworks, UiO-66, substituent effect, DFT calculations

## Abstract

The nature of organic linker substituents plays an important role in gas sorption and separation as well as in catalytic applications of metal–organic frameworks. Zirconium-based UiO-66 is one of the most tunable members of this class of materials. However, the prediction of its properties is still not a fully solved problem. Here, the infrared spectroscopic measurements using highly sensitive CO probe molecules, combined with DFT calculations, are used in order to characterize the performance of different acidic sites caused by the presence of different organic linker substituents. The proposed model allowed differentiation between various active sites over the UiO-66 and clarification of their behavior. The experimental IR bands related to CO adsorption can be unambiguously assigned to one type of site or another. The previously undescribed highly red-shifted band is attributed to CO adsorbed on coordinatively unsaturated zirconium sites through an O atom. The results confirm the lower and higher Lewis’s acidity of coordinatively unsaturated Zr sites on linker defects in the UiO-66 structure when electron-withdrawing and electron-donating groups are, respectively, included in a terephthalate moiety, whilst the Brønsted acidity of zirconium oxo-cluster remains almost unchanged.

## 1. Introduction

Metal–organic frameworks (MOFs) belong to a class of functional solid materials, and their properties can be tuned for a wide range of applications [1]. They can be fabricated into a countless variety of structures with diverse surface chemistries and pore widths and shapes [2,3]. This is because the structures of MOFs comprise both organic and inorganic parts which are bonded via strong chemical bonds with the formation of open crystalline frameworks. Recently, the concept of bimetallic metal–organic frameworks was developed [4], which further expanded the opportunities to synthesize such materials with desired properties. However, the main difference between MOFs and inorganic crystalline materials is the opportunity for chemical functionalization through the modification of organic linkers.

**Figure 1:**
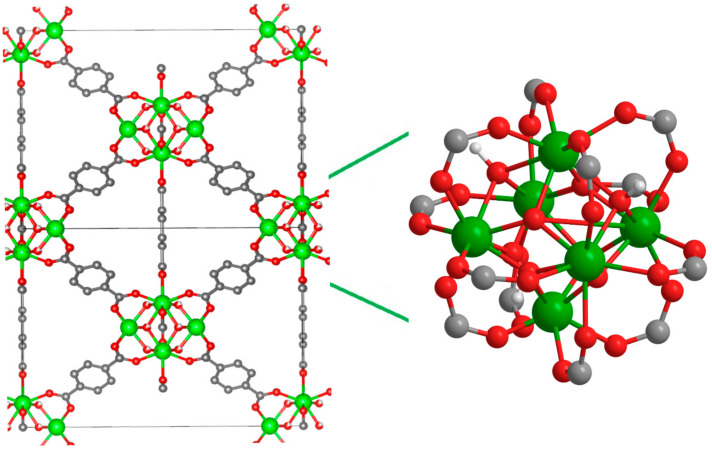
Representation of UiO-66 structure. Color code: Green, red, gray, and white balls correspond to zirconium, oxygen, carbon, and hydrogen atoms, respectively.

UiO-66 has been attracting wide research interest due to its high thermal and solvothermal stability and relatively simple synthesis in a wide range of conditions with potential scalable techniques and the high tunability of physical–chemical properties [5]. It was first synthesized by Lillerud’s group from ZrCl_4_ and terephthalic acid [6]. In its most stable hydroxylic form, the UiO-66 crystal comprises a structure with a face-centered cubic unit cell with *fm-3m* symmetry. The six zirconium atoms in the UiO-66 node are present at the vertices of an octahedron, with four oxygen and four hydroxyl groups protruding out of the eight faces. The carboxylate groups of terephthalates bridge the edges of the nodes to form a Zr_6_O_4_(OH)_4_(CO_2_)_12_ cluster (Figure 1) [7,8]. In a pristine structure, each node is coordinated with twelve terephthalate ligands. However, when one or several organic bridges are missing, the so-called “missing linker defects” appear. This leads to the formation of coordinatively unsaturated Zr sites (Zr_CUS_).

Vermoortele et al. [9] demonstrated in 2012 that the concept of the electronic modulation of the active site could be transferred to catalytic MOFs, and a number of works were devoted to the effect of terephthalic acid substituents on the activity of UiO-66 in Lewis acid-catalyzed reactions [10,11,12,13].

The inductive effect of functional groups in the linker was shown to have an influence on the Lewis acidity of the Zr active site and, as a consequence, the catalytic activity of X-UiO-66 [9,10]. Recently, some of us demonstrated that the substituent’s nature has a significant effect on the catalytic activity of X-UiO-66 in the oxidation of propylene glycol with hydrogen peroxide [14]. Yin et al. found the effect of a substituent in a UiO-66-X-based electrically driven self-cleaning membrane [15]. Moreover, organic linker functionalization was demonstrated to be of importance for gas sorption [16,17,18], the removal of water pollutants [19], and optical properties of UiO-66 [20].

At the same time, the development of adequate theoretical models, which could be used for DFT calculations with moderate time and computational costs, can reflect the substituent nature effect on different MOF properties. This is a topical challenge in view of the growing field of the MOF application. The prediction and tuning of its properties prior to synthesis is an important step for more sustainable and green chemistry. However, the structure–property relationships are still not properly understood, which leads to difficulties in identifying the promising MOF candidates with the optimal set of electronic properties [21].

Different spectral methods are used to study the nature of acid–base sites, in addition to the specificity of the catalytic effect of MOFs at the atomic molecular level and to predict their catalytic properties [22]. One of the main probe molecules used to study the Lewis acid sites in MOFs is carbon monoxide. CO is widely employed as a highly effective IR probe because the vibrational frequency of the C-O bond in the adsorbed molecule strongly depends on the electronic character of the site to which it is bound [23,24]. Wiersum et al. [25] were the first to investigate the CO adsorption on unsubstituted UiO-66 (H-UiO-66). They described three bands in the C-O region after CO interaction with Zr-MOF and attributed them to CO interaction with weak Brønsted sites, Zr Lewis acid sites, and physisorbed CO. Later, Driscoll et al. [26] tried to make the atomic level characterization and quantification of defects using IR spectroscopic studies with a carbon monoxide probe. Using DFT calculations, they also revealed that the adsorption of CO on the coordinately unsaturated Zr sites and μ_3_-OH blue shifts the C-O stretch. The same research group showed that CO interaction with μ_3_-OH groups in non-defective UiO-66 could be realized through the O atom of the CO molecule and it is red-shifted [27]. Cirujano and Llabrés i Xamena showed that Zr^4+^ sites in a hydrated sample strongly polarized the coordinated water molecule, leading to new Brønsted acid sites [28]. The C-O vibration in hydrogen-bonded CO with this water molecule led to the appearance of the band at 2142 cm^−1^, which was absent in the spectrum of samples activated at 200 °C.

Chakarova et al. [29] compared acid and basic sites in UiO-66 and NH_2_-UiO-66 using different probe molecules, particularly CO, in a FT-IR study. They concluded that the hydroxyls of NH_2_-UiO-66- are slightly more acidic in comparison with bare UiO-66. However, they did not try to build a theoretical model describing the reasons for such transformations.

In the present work, we have applied a strategy connected with the characterization of the acidity of metal-oxide nodes in the UiO-66 structure, depending on the nature of the substituent in the terephthalate linker, using the combination of IR, adsorbed CO, and DFT calculations. We found the simple theoretical model to demonstrate the influence of the substituent nature on the electronic properties of Zr active sites obtained through missing linker defects.

## 2. Results and Discussion

### 2.1. CO Adsorption on H-UiO-66

Wiersum et al. showed that CO hardly adsorbed on UiO-66 at room temperature [25]. This is not surprising, because the Zr^4+^ cation has the configuration of a noble gas and does not have *d* electrons in its electron shell for the formation of a *π* bond; therefore, its interaction with ions or neutral molecules is mostly electrostatic. Thus, due to low dipole moment, CO is not the preferred ligand for Zr^4+^ [23]. Taking this into account, the adsorption experiments were carried out at 77 K after the dehydroxylation of X-UiO-66 samples at 473 K (see Section 3). 

**Figure 2:**
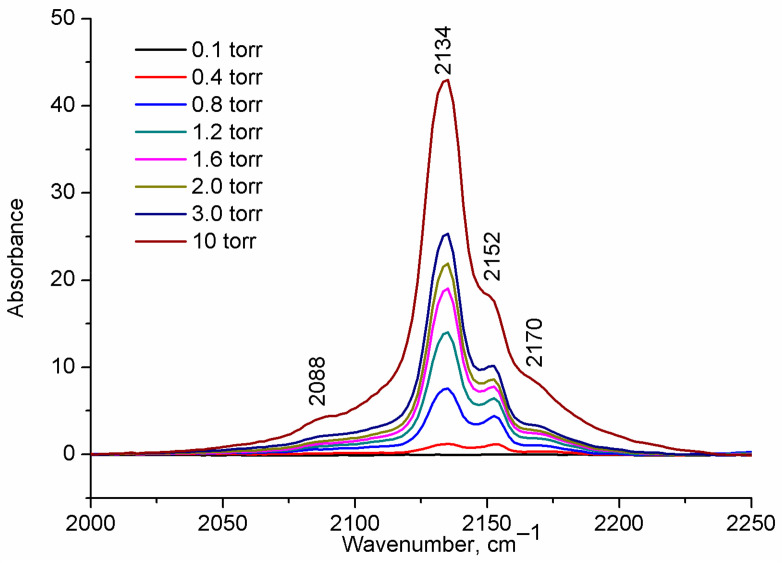
FT-IR spectra of CO adsorbed at 77 K on H-UiO-66.

Figure 2 shows the FT-IR spectra for H-UiO-66.

The most prominent band at 2134 cm^−1^, with a rapidly growing intensity as the CO pressure increases, is slightly red-shifted relative to the gaseous CO (2143 cm^−1^ [30]) and can be assigned to physisorbed CO [25,28,29]. 

**Figure 3:**
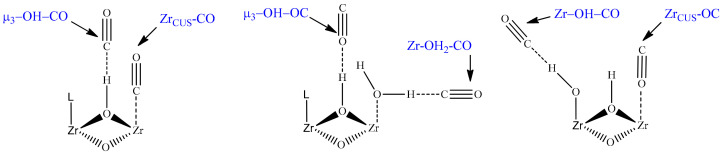
Representation of the main CO adsorption sites on UiO-66.

A sharp band at ~2152 cm^−1^ is commonly attributed to CO that is H-bonded with μ_3_-OH hydroxyls through a carbon atom [25,26,27] (see Table 1 and Figure 3) and is accompanied by a shift of the hydroxyl vibration band (∆ν(OH) ≈ 83 cm^−1^), as was previously shown in Ref. [25]. A highly blue-shifted band (relative to gas-phase CO) at 2170 cm^−1^ is assigned to CO in a dative bond with coordinatively unsaturated Zr sites (Zr_CUS_) at a missing linker defect (σ bond through C atom) [26]. The evidence of attribution of this band to CO interaction with Lewis acid sites in UiO-66 is the absence of another shift of band in the hydroxyl vibration region [25], the increase in its intensity with the increase in the defectiveness of UiO-66 [26], and its disappearance in the spectrum of sample activated only at 60 °C [28] and saturated D_2_O [26]. Moreover, the DFT calculations of Driscoll et al. [26] showed that CO adsorption directly on coordinatively unsaturated Zr sites led to a higher blue shift of C-O vibration band in comparison with that for CO hydrogen bonded to a μ_3_-OH (Table 1). In the experimental spectrum, the intensity of this band is lower than that of the band at 2152 cm^−1^, due to a lower number of defects in comparison with the number of μ_3_-OH groups (four per one zirconium oxo-cluster) in the sample (Figure 1). 

One more relatively intensive band was highly red-shifted (2088 cm^−1^) and was not described previously. Our assumptions on the nature of its appearance will be discussed below. Another band at ~2142 cm^−1^, which corresponds to ν_(C-O)_ mode in an H-bonded adduct of CO with water molecules coordinated on defects [28] (see Zr-OH_2_-CO in Figure 3), was not observed in our experiment due to the high activation temperature (473 K) of the samples before the experiment.

**Figure 4:**
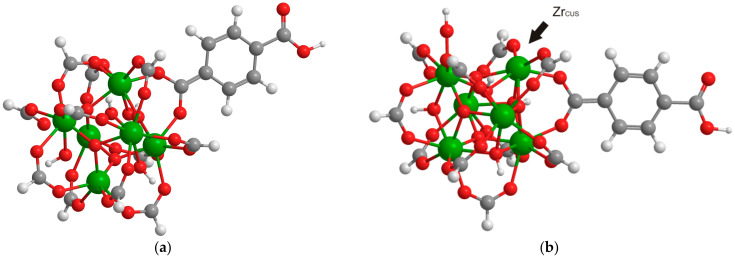
Example of model used comprising a UiO node with a single linker. Color code: Green, red, gray, and white balls correspond to zirconium, oxygen, carbon, and hydrogen atoms, respectively. (**a**) Non-defective, (**b**) With a single linker defect.

The DFT calculations were performed using relatively small cluster models with only one terephthalate linked with the Zr_6_O_4_(OH)_4_ node. The remaining 11 linkers were replaced with formates in order to save the coordination number and the charge of all Zr atoms in the node (Figure 4a). In order to modulate the CO interactions with the sites formed through linker vacancies, a single formate linker was removed from the node with the formation of the coordinatively unsaturated Zr atom linked with terephthalate (Figure 4b). 

**Figure 5:**
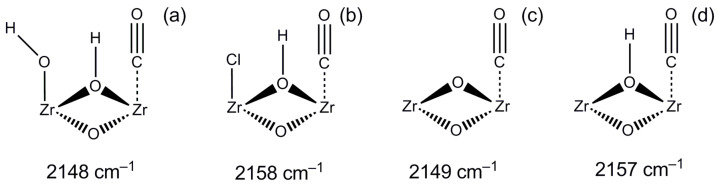
Vibration frequencies of CO adsorbed on Zr_CUS_ sites of H-UiO-66 with different counterions (OH^–^(**a**), Cl^–^(**b**), H^+^ removal-compensated (**c**), uncompensated (**d**)).

One of the most debated topics concerning the UiO-66 structure is the nature of particles on Zr defect sites [31]. To a certain extent, their nature is determined by the reaction medium in which the synthesis of MOFs takes place. Taking into account that in the present work, the X-UiO-66 samples were synthesized from ZrO(NO_3_)_2_, the corresponding organic linker in the presence of water, HCl (modulator), and N,N-dimethylformamide (solvent), one can assume that there are several species that could be coordinated to Zr sites. The formation of linker vacancies through the removal of the negatively charged linker leads to the appearance of a positive charge on a zirconium oxo-cluster. Thus, negatively charged OH^-^ and Cl^-^ are the main candidates for compensation of the excess positive charge. Moreover, water and N,N-dimethylformamide (DMF) molecules can also be coordinated to defect sites and remain in the UiO-66 crystal structure after synthesis. It is noteworthy that the presence of only water and DMF molecules cannot compensate the excess charge; therefore, they can only be present as “auxiliary” molecules, along with anionic particles. However, the removal of the proton from one μ_3_-OH in Zr_6_O_4_(OH)_4_ can also lead to charge compensation. Both described models were calculated for the defect cluster containing Zr_CUS_ (Figure 5). The presence of these counterions is consistent with the earlier developed assumptions [32,33,34]. The absence of neutral DMF and H_2_O molecules on the neighboring Zr atom was provided by the pretreatment conditions (see above) and was taken into account during the calculations. Additionally, the system with uncompensated charge was also calculated. 

Interestingly, in the OH^—^ and H^+^ removal-compensated models (Figure 5a,c), the frequencies of vibrations of CO coordinated on Zr_CUS_ are more red-shifted (2148 and 2149 cm^−1^) in comparison with those for the Cl-containing model (Figure 5b) and the uncompensated model (Figure 5d) (2158 and 2157 cm^−1^, respectively). The latter two models are consistent better with the experimental data (see Figure 2 and Table 1). A similar trend is also observed for NH_2_-UiO-66 (Figure S1 in Supporting Information (SI)).

The experimental IR bands related to CO adsorption can be unambiguously assigned to one type of site or another. Good correlation between predicted and experimental vibration frequencies is achieved (see Table 1 and Figure S2). The calculated CO vibrational frequencies correlate comparably with experimental data as well as with the previous values obtained by other groups (Table 1), although the present theoretical model is less complicated. It is noteworthy that the calculated vibrational frequency for the isolated CO molecule is slightly lower than the experimental value for gas phase CO (2110 cm^−1^, whilst the experimental value was 2143 cm^−1^). Thus, all other calculated CO vibration frequencies are slightly lower than the experimental ones, although the relative shift for the frequencies of CO adsorbed on different UiO-66 sites is consistent with the experimental spectrum (Table 1 and Figure S2 in SI). The vibrational frequencies of CO adsorbed on μ_3_-OH are calculated using the non-defective model (Figure 4a) due to a higher fraction of μ_3_-OH sites that are not bonded with the Zr_CUS_.

The calculations confirm the attribution of the main bands at ~2100, 2140, and 2170 cm^−1^ to vibrations of CO adsorbed on μ_3_-OH (Figure S3 in SI) and Zr_CUS_ made by other research groups (see Table 1). Moreover, we firstly confirm the assumption of Cirujano and Llabrés i Xamena [28] about the acidity of the water molecule, which occupied one of the Zr atoms on defect sites. In the model used, the neighboring Zr atom was occupied by a negatively charged OH^–^ group (Figure S4 in SI), which is expected to be present in a rehydroxylated sample. The calculated value of CO vibration frequency (2124 cm^−1^) is slightly lower in comparison with the experimental value obtained in Ref. [28]. However, it is in the overall trend that the lower calculated CO vibration frequencies and the corresponding shift located between the shifts were attributed to μ_3_-OH···OC and μ_3_-OH···CO (see Table 1).

Although the interaction of CO with μ_3_-OH through an oxygen atom was previously described [27], the assumptions about the possibility of CO adsorption on Zr_CUS_ through an O atom were not made. At the same time, it is known that the position of the corresponding band for CO adsorption on alkaline earth cations is below the CO gas-phase stretching frequency and is placed in the region around 2092 cm^−1^ [23]. The appearance in the experimental spectrum of the band at 2088 cm^−1^, which was not previously described, allowed us to assume that it could be attributed to the Zr_CUS_-OC case (see Figure 3). The calculated value (2062 cm^−1^) is also highly red-shifted in comparison with the stretching vibration of CO freely rotating in a gas, in agreement with the experimental value (2088 cm^−1^).

### 2.2. Effect of Substituent

**Figure 6:**
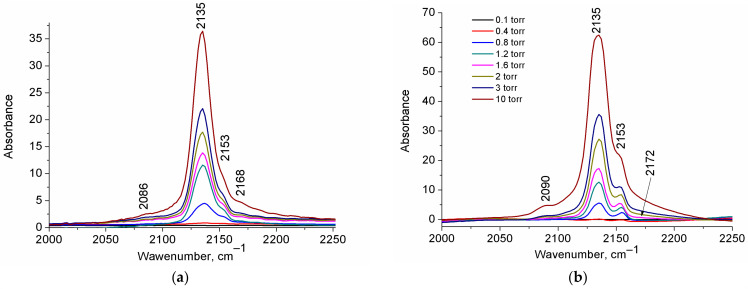
FT-IR spectra of CO adsorbed at 77 K on NH_2_-UiO-66 (**a**) and NO_2_-UiO-66 (**b**).

Figure 6 shows the FT-IR spectra of CO adsorbed on NH_2_-UiO-66 and NO_2_-UiO-66. Both spectra demonstrate the presence of the same bands as the corresponding spectrum for CO adsorbed on H-UiO-66 (Figure 2). While the bands corresponding to physisorbed CO and bonded with μ_3_-OH feature the same wavenumber (2135 and 2153 cm^−1^, respectively), the band corresponding to CO adsorbed on coordinatively unsaturated sites acting as Lewis acids demonstrate red and blue shifts for NH_2_- and NO_2_-UiO-66, respectively. These shifts unambiguously confirm higher and lower electron density around Zr_CUS,_ respectively, for NH_2_- and NO_2_-substituted models in comparison with the H-UiO-66. It is in agreement with the Hammett constants (*σ_p_*) for the mentioned substituents (−0.66 and 0.78 for NH_2_- and NO_2_-, respectively) corresponding to their electron-donating and electron-withdrawing properties [35].

Thus, the nature of the organic linker substituent has a significant impact on the Lewis acidity of Zr-UiO-66 and does not change the acidity of the OH groups presented in its structure after evacuation at 473 K. These observations are consistent with the influence of the organic linker substituent in Zr-UiO-66 on Lewis acid-catalyzed reactions, where the presence of electron acceptors in terephthalate linkers leads to a significant increase in the reaction rate [9,10].

Table S1 shows the bands at the pressure of 10 torr. The overall concentration of CO complexes with Zr_CUS_ and μ_3_-OH increases with the increase in the surface area (see Table S2 in SI). The concentration of CO molecules H-bonded with μ_3_-OH is similar for NH_2_- and NO_2_-containing samples (Table S1 in SI); however, the concentration and relative ratio of Zr_CUS_-CO and Zr_CUS_-OC vary significantly for different samples. Interestingly, the ratio of integral intensities of the bands corresponding to Zr_CUS_-CO and Zr_CUS_-OC is similar for NH_2_- and H-UiO-66 but strongly differs from the same ratio for NO_2_-UiO-66 (Table S1 in SI). In order to realize the reasons, we paid attention to the nature of CO interaction with metal ions. As mentioned above, the CO adsorption on the Zr^4+^ ion is realized via electrostatic and *σ* bond interaction. Bonding of the 4*σ* MO orbital located at the oxygen atom of CO is much more stable than the slightly antibonding 5*σ* MO located at the C atom that donates electron density to metal ions in the case of bonding through the carbon atom. Therefore, the contribution of the *σ* Zr-OC bond in the isocarbonyls is smaller than in the case of the C-bonded species [23]. Thus, the complexes with O-bonded CO are expected to be formed with the cations for which electrostatic interaction dominates. This may explain the predominant adsorption of CO on Zr_CUS_ due to lower electron density on these sites in NO_2_-UiO-66, in comparison with other MOFs discussed here.

**Figure 7:**
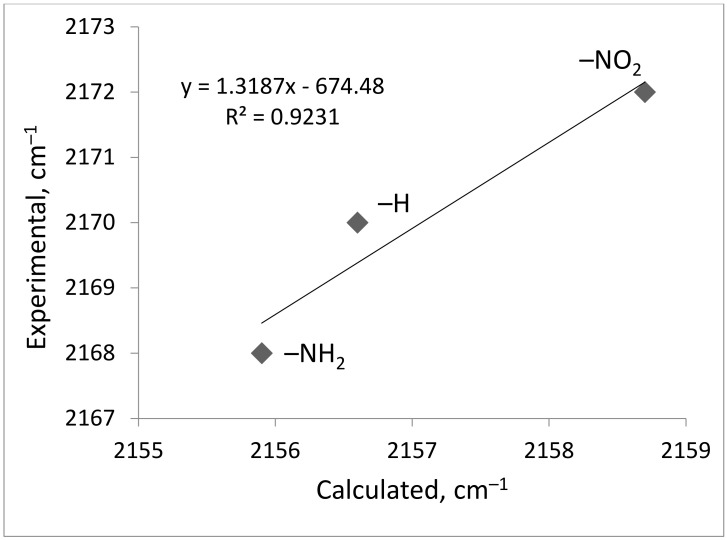
Correlation of calculated and experimental wavenumbers for vibrations of Zr_CUS_-CO for X-UiO-66 (X = H-, NH_2_-, NO_2_-).

DFT calculations confirm the blue and red shift of vibrations of CO adsorbed on Zr_CUS_ of NO_2_- and NH_2_-UiO-66, respectively. This is applied to both Zr_CUS_-CO and Zr_CUS_-OC (Table 2). The calculated vibration frequency of CO adsorbed on μ_3_-OH is not altered by the presence of a substituent (Table 2), which supports the assumption about the influence of the substituent’s nature only on Zr-UiO-66 Lewis acidity. The calculated relative shifts are consistent with the experimental data (Figure 7). The relatively lower shift for NH_2_-UiO-66, in comparison with the NO_2_-UiO-66 relative vibration of Zr_CUS_-CO in UiO-66, can be explained by the lower redistribution of electronic density on the reaction site by the presence of NH_2_- in the aromatic system in comparison with the NO_2_ group (see the corresponding Hammett constants above). 

In the present work, we attempted to predict the effect of the organic linker substituent’s nature on the acidity of Zr-UiO-66 active sites. For this purpose, we chose the model where CO molecules do not interact directly with the substituent. However, it is obvious that the specific interaction of the adsorbed molecules with the substituent group during gas storage, gas separation, or catalysis can play an important role. Thus, the change in the orientation of these groups (away from the defect or towards it), and their positions (orto- or meta-), can help to reveal the reasons for the substituent effect on MOF properties. The research model developed in the present work can be used in MOF design strategies and the fine-tuning of UiO-66 properties for a wide range of tasks in the most facile and effective way.

## 3. Materials and Methods

All of the reagents used were obtained from commercial suppliers and were used without further purification.

The X-UiO-66 materials were synthesized and characterized as described in Ref. [14]. Then, 3 mmol of therephthalic acid (or NO_2_-/NH_2_-substituted one) was dispersed in N,N-dimethylformamide (DMF), and then, hydrochloric acid (99 mmol for H- and NO_2_-UiO-66) was added. To synthesize the NH_2_-UiO-66, a higher HCl amount was required (132 mmol). The obtained mixture was heated at 120 °C for 24 h (H- and NO_2_-UiO-66) or 12 h (NH_2_-UiO-66) in a Teflon-lined stainless-steel autoclave. The obtained solids were sequentially washed with DMF and ethanol.

The IR spectra of the CO adsorbed on the surface of X-UiO-66 were recorded using the Shimadzu-8300 spectrometer (Shimadzu Scientific Instruments, Kyoto, Japan) in the range of 400–6000 cm^−1^, with a spectral resolution of 4 cm^−1^ in the absorbance scale with an accumulation of 200 scans. Samples were prepared in the form of tablets weighing 15–200 mg/cm^2^. Prior to the analysis, the samples were pretreated under 10^−3^ mm Hg vacuum at 473 K for 1 h to achieve the almost complete removal of water molecules, free acid, and DMF. At the same time, μ_3_-OH hydroxyls were present up to 573 K, and the zirconium oxo-cluster was still in hydroxylated form at 473 K. CO adsorption was carried out by injecting the cell with the sample at 77 K after the dehydroxylation of X-UiO-66 samples at 473 K. The CO was adsorbed in portions at pressures from 13 to 200 Pa. The maximum CO pressure at which concentrations were calculated was 1300 Pa. The background spectrum was recorded before the CO adsorption.

## 4. Computational Details

The DFT calculations were carried out using the Gaussian’09 program package (Revision C.01) (Wallingford, CT, USA) installed on the SKIF “Cyberia” supercomputer of Tomsk State University [36]. The model of the UiO-66 node was excerpted from the crystallographic data [37]. The node was formed using six Zr atoms surrounded by oxygen atoms, HCOO moieties, and a single terephthalate linker. This allowed the modeling of various defect sites (including coordinatively unsaturated Zr sites) at moderate time and computational costs. The long-range correlation functional wB97XD [38] utilizing empirical dispersion and long-range correlations was used. The geometries of the systems considered were fully optimized using the quasi-relativistic effective core pseudopotential Lanl2DZ for Zr atoms [39] and the split valence 6-31G basis set for C, H, O, N, and Cl atoms [40]. Testing of the wB97XD/6-31G level of theory for an isolated CO molecule provided high consistency of the calculated IR band (the calculated value for CO stretching was 2110 cm^−1^, whilst the experimental value was 2143 cm^−1^ [30]). All structures were fully optimized. The convergence criterion of −1.0D-08 (a.u.) was employed. The IR spectrum was modeled to check the nature of the stationary point. The absence of imaginary frequencies confirmed the achievement of a stationary state.

## 5. Conclusions

Infrared spectroscopy of adsorbed CO accompanied by DFT calculations provided evidence of the effect of the organic linker substituent nature on the performance of active sites in the UiO-66 structure. A relatively small theoretical model showed high predictive ability towards the acidity of MOF sites. All bands in the C-O vibrations region were fully characterized and can be unambiguously assigned to one type of site or another. The highly red-shifted bands in the region of 2086–2090 cm^−1^ were first observed for H-UiO-66 and attributed to CO adsorbed on zirconium coordinatively unsaturated sites through an O atom. The calculations confirmed the lower and higher Lewis acidity of Zr coordinatively unsaturated sites on linker defects in the UiO-66 structure featuring electron-withdrawing and electron-donating groups in the therephthalate moiety, respectively, whilst the Brønsted acidity of the Zr oxo-cluster remained almost unchanged. The model developed can be used in the design and fine-tuning of the UiO-66 features for a wide range of applications.

## Figures and Tables

**Table 1:** Main FT-IR bands in C-O region of CO adsorbed on H-UiO-66.

Centers of CO Adsorption (See Figure 3)						Reference
Zr_CUS_-CO	μ_3_-OH-CO	μ_3_-OH–OC	Zr-OH_2_-CO	Zr_CUS_-OC	Physisorbed	
2172	2155	-	-	-	2136	[25]
2180 (2194)	2154 (2170)	-	-	-	2148	[26]
-	2152 (2179)	2124 (2121)	-	-		[27]
2180	2153	2126	-	-	2136 and 2132	[29]
2178	2153–2154	-	2142	-	2137–2138	[28]
2170 (2157)	2152 (2140)	2100 (2087)	- (2124)	2088 (2066)	2134	This work

The values of the corresponding band wavenumbers are given in cm^−1^. Calculated values are given in parentheses.

**Table 2:** A comparison of experimental and calculated wavenumbers of selected FT-IR bands in C-O region of CO adsorbed on X-UiO-66 (X = NH_2_-, H-, NO_2_-).

Sample	CO Adsorption Sites (See Figure 3)		
	Zr_CUS_-CO	μ_3_-OH-CO	Zr_CUS_-OC
NH_2_-UiO-66	2168	2153	2086
	(2156)	(2140)	(2064)
H-UiO-66	2170	2152	2088
	(2157)	(2141)	(2066)
NO_2_-UiO-66	2172	2153	2090
	(2159)	(2140)	(2068)

The experimental values for the corresponding band’s wavenumber are given in cm^−1^. Calculated values are given in parentheses.

## Data Availability

Data available on request.

## Supplementary Materials

The following supporting information can be downloaded at: https://www.mdpi.com/article/10.3390/ijms241914893/s1, Figure S1: Vibration frequencies of CO adsorbed on ZrCUS sites of NH2-UiO-66 with different counterions; Figure S2: Correlation of the calculated and experimental vibration frequencies of ZrCUS-CO; Figure S3: Calculated geometries of CO adsorption on μ3-OH sites in UiO-66 node. Color code: Green, red, gray, and white balls correspond to zirconium, oxygen, carbon, and hydrogen atoms, respectively; Figure S4: Calculated geometries of CO H-bonded with water molecules in the UiO-66 node with one linker defect (Zr-OH2-CO). Color code: Green, red, gray, and white balls correspond to zirconium, oxygen, carbon, and hydrogen atoms, respectively; Table S1: Bands integral intensities at the pressure of 10 torr; Table S2: Textural characteristics of UiO-66-X.
