# Supplementary material for: Effect of Linker Substituent Nature on Performance of Active Sites in UiO-66: Combined FT-IR and DFT Study
Source: Int J Mol Sci. 2023 Oct 4;24(19):14893. doi: 10.3390/ijms241914893 (PMC10573255; doi:10.3390/ijms241914893)
Supplement: Supplementary file 1 [file ijms-24-14893-s001.zip › ijms-2628390-supplementary.pdf]

International Journal of *Molecular Sciences*

MDPI

Supporting Information

Effect of linker substituent nature on performance of active sites in UiO-66: Combined FT-IR and DFT study

Viktoriia V. Torbina [1], Mikhail A. Salaev [1], Evgeniy A. Paukshtis [2], Leonarda F. Liotta [3] and Olga V. Vodyankina [1,*]

[1] Tomsk State University, Lenin ave., 36, Tomsk, 634050, Russia

[2] Boreskov Institute of Catalysis, SB RAS, Ak. Lavrentieva ave., 5, Novosibirsk, 630090, Russia

[3] Institute for the Study of Nanostructured Materials (ISMN), National Research Council (CNR), 90146 Palermo, Italy

* Correspondence: leonardafrancesca.liotta@cnr.it, vodyankina_o@mail.ru

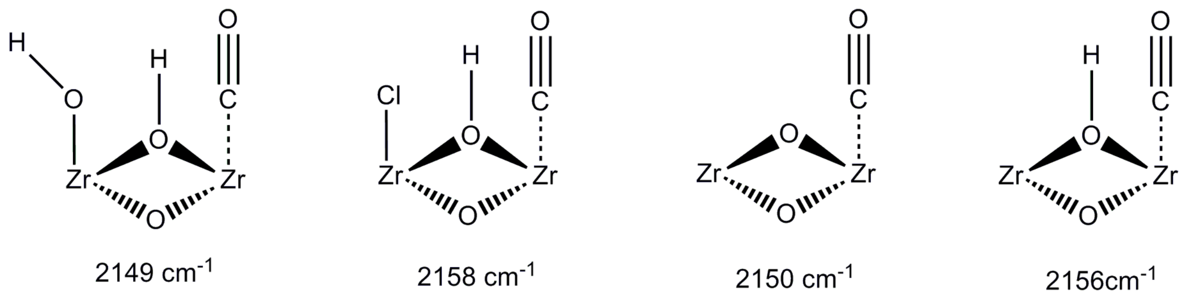

Figure S1. Vibration frequencies of CO adsorbed on Zr_{CUS} sites of NH_2-UiO-66 with different counterions.

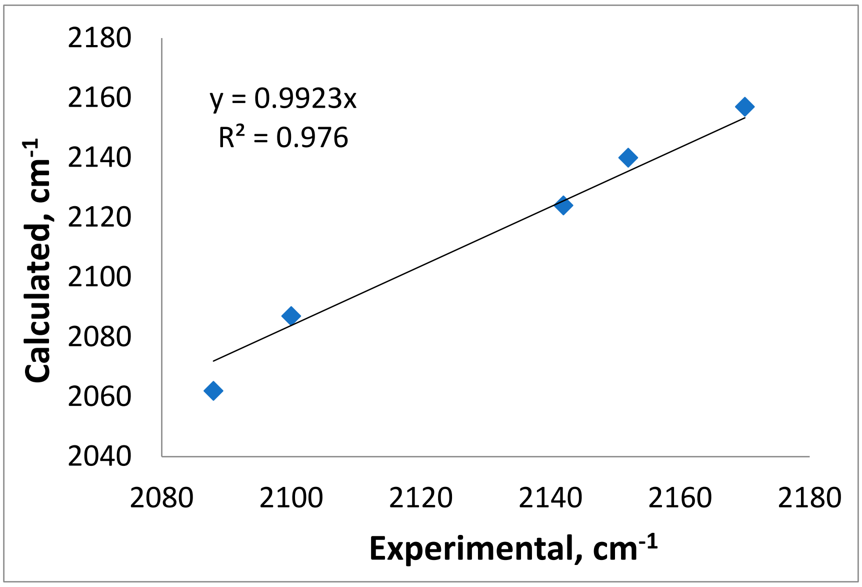

Figure S2. Correlation of the calculated and experimental vibration frequencies of Zr_{CUS}-CO.

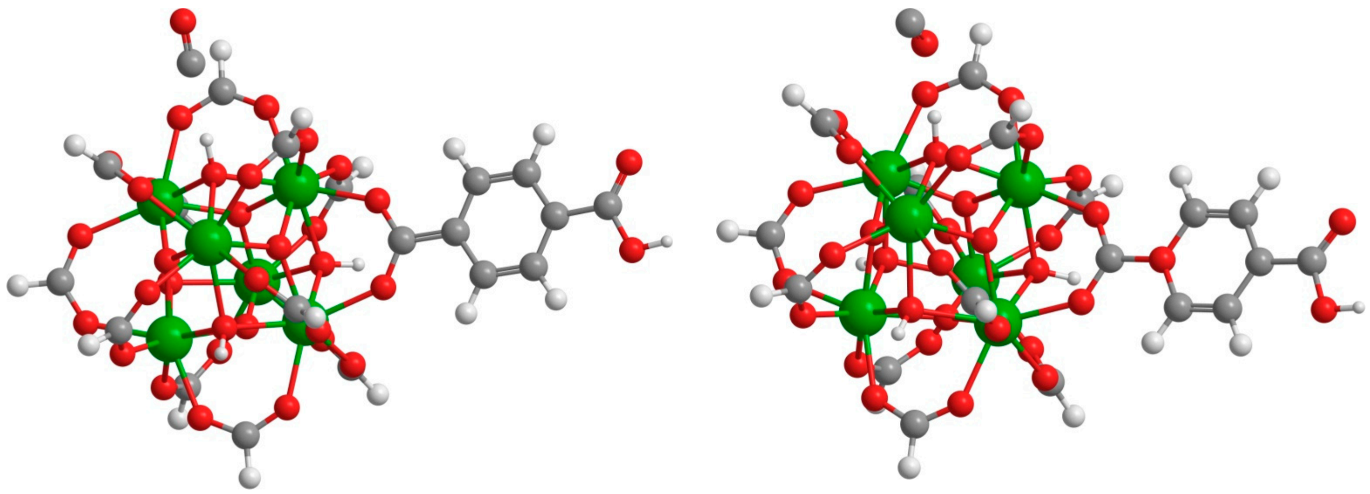

(**a**) μ_3-OH-CO (**b**) μ_3-OH-OC

Figure S3. Calculated geometries of CO adsorption on μ_3-OH sites in UiO-66 node. Color code: Green, red, gray, and white balls correspond to zirconium, oxygen, carbon, and hydrogen atoms, respectively.

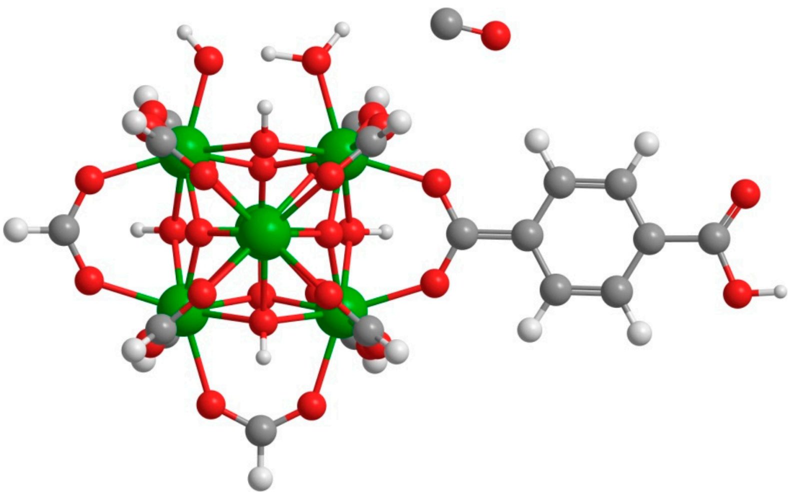

Figure S4. Calculated geometries of CO H-bonded with water molecule in the UiO-66 node with one linker defect (Zr-OH_2-CO). Color code: Green, red, gray, and white balls correspond to zirconium, oxygen, carbon, and hydrogen atoms, respectively.

Table S1. Integral intensities of bands at the pressure of 10 torr.

Sample	Zr_{CUS}-CO	μ_3-OH-CO	Physisorbed	Zr_{CUS}-OC
NH_2-UiO-66	40	83	560	50
H-UiO-66	100	94	710	120

NO_2-UiO-66	50	83	1220	120

Table S2. Textural characteristics of UiO-66-X.

**Sample**	**S_{BET}, m^2/g**	**V_{pores}, cm^3/g**
NH_2-UiO-66	719	0.29
H-UiO-66	1204	0.45
NO_2-UiO-66	691	0.28

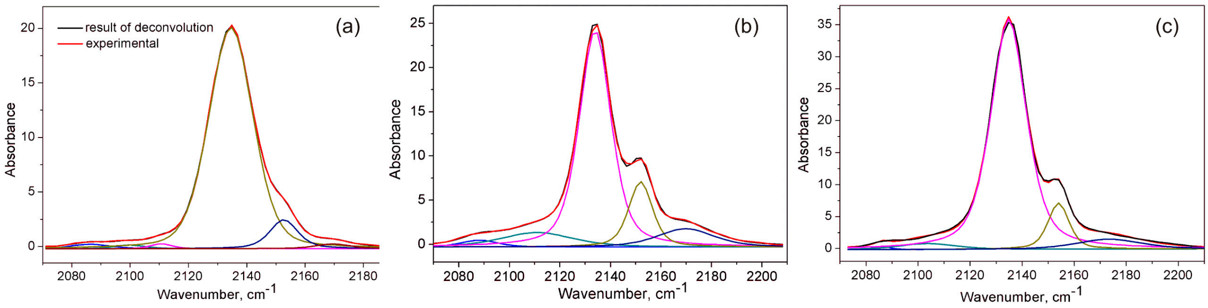

Figure S5. Deconvolution results for FT-IR spectrum of adsorbed CO on NH_2- (a), H- (b), and NO_2-UiO-66 (c).

Cartesian coordinates for nondefective cluster model

C 0.6376 2.6841 -2.4233

C 3.1636 -0.1439 -0.3403

C 0.4116 -3.7829 -2.0533

C 1.3366 3.0691 1.9367

C -0.7394 -0.0789 4.7307

C -3.6544 -3.5599 -1.7303

C -5.7644 -0.0499 0.7537

C 1.1116 -3.3009 2.6247

C -3.7544 2.6571 -1.9703

C -3.2534 -3.2349 3.1217

C -3.3924 3.1141 2.5037

O -0.1694 3.1051 -1.5083

O 0.8076 1.4501 -2.7343

O 0.6456 -2.5859 -2.4923

O 2.3586 -0.3769 -1.3973

O 0.6706 -1.7689 0.1407

O -1.3334 0.6218 -1.1387

O 0.0366 0.7501 -0.0653

O -1.4694 -1.4889 -1.0183
O 2.5906 -0.1369 0.8597
O -0.2054 -4.0719 -0.9653
O -1.0494 1.3161 2.1667
O 1.4676 -2.0649 2.6837
O 1.5666 1.8651 2.3017
O 0.3256 -0.0509 4.0087
O -1.1374 -1.2039 1.8087
O -3.3264 -1.6809 0.6277
O -4.0064 1.4121 -2.2093
O -3.9184 -2.3209 -2.0513
O -5.3104 -0.2319 -0.4443
O -2.6884 0.7951 0.3047
O -2.5494 -3.7349 2.1627
O 0.1626 -3.7709 1.8827
O -2.8894 -3.9309 -0.7743
O -2.5934 3.4681 1.5457
O -2.7984 3.0791 -1.2213
O 0.3276 3.4381 1.1997
O -1.9554 -0.0339 4.2857
O -5.0494 -0.0209 1.8247
O -3.5164 -1.9819 3.2817
O -3.5644 1.9091 2.9107
Zr 0.2716 -0.5179 -1.7063
Zr -3.2414 -0.4299 -1.2513
Zr -1.2684 2.3561 0.2027
Zr 0.7176 -0.1669 1.7927
Zr -1.3244 -2.8529 0.5307
Zr -2.8484 -0.1189 2.2607
H -1.4061 1.1473 -1.9239
H 1.5076 -2.2719 0.1197
H -0.9214 1.8691 2.9597
H -4.1244 -2.2179 0.7867
C 4.5586 0.0861 -0.4803
H 1.6626 -4.0149 3.2467
H -0.6114 -0.1319 5.8177
H -3.9564 3.9101 3.0027
H -6.8464 0.0841 0.8647
H -3.6724 -3.9329 3.8557
H 0.7776 -4.6169 -2.6643
H 1.2016 3.4401 -2.9803
H -4.1424 -4.3339 -2.3343

H 2.0256 3.8541 2.2657
H -4.3954 3.4051 -2.4503
C 7.3556 0.5841 -0.6163
C 5.3366 0.3141 0.7157
C 5.2376 0.1141 -1.7633
C 6.6316 0.3591 -1.7863
C 6.6916 0.5571 0.6547
C 8.8746 0.8751 -0.6983
O 9.4146 1.2071 0.4217
O 9.3896 0.7561 -1.8703
H 4.8116 0.2881 1.6657
H 7.1916 0.3791 -2.7193
H 7.3026 0.7441 1.5317
H 4.6676 -0.0459 -2.7163
H 10.4836 1.2671 0.4727
C -1.7264 -0.7669 -3.6533
O -0.5154 -0.7099 -3.2003
O -2.7944 -0.6479 -2.9433
H -1.7894 -0.8419 -4.8503
